# Influence of insole material density in the stability of patients with prosthetic unilateral transtibial amputation

**DOI:** 10.1038/s41598-022-11564-3

**Published:** 2022-05-12

**Authors:** Nuria Sarroca, María José Luesma, José Valero, María Pilar del Caso, Cristina Alonso, Jorge Calleja, Tania Lorenzo, Javier Bayod, Manuel Lahoz

**Affiliations:** 1 Private Practice, Madre Vedruna 14 bajo derecho, 50008 Zaragoza, Spain; 2 Department of Human Anatomy and Histology, University of Zaragoza, Calle Domingo Miral s/n, 50009 Zaragoza, Spain; 3 Private Practice, Coso 55, 50001 Zaragoza, Spain; 4 Private Practice, Baltasar Gracián 6, 50005 Zaragoza, Spain; 5 Daroca Health Centre (Calatayud Sector), Luchente s/n, 50360 Zaragoza, Daroca Spain; 6 Department of Hospital Medicine, San Carlos Clinical Hospital, Prof Martín Lagos S/N, 28040 Madrid, Spain; 7 Department of Mechanical Engineering, University of Zaragoza, 50009 Zaragoza, Spain

**Keywords:** Medical research, Neurology, Rheumatology

## Abstract

People with lower limb amputation present greater displacements of their centre of gravity in a static situation than able-bodied individuals, as they depend on visual information to a greater extent, which implies an altered stability pattern. The efficacy of different hardness of plantar support to help maintain stability has not yet been determined. The aim of the present study is to assess stability in people with unilateral transtibial amputation with prosthesis in a static situation with insoles of different degrees of hardness and visual conditions with respect to the able-bodied population. For this purpose, 25 patients with amputation and 25 able-bodied individuals were included in both groups, postural stability was assessed by stabilometry. This assessment was carried out under normal conditions (on the floor of the dynamometric platform with eyes open), and under altered conditions (with the interposition of different materials such as plantar support: rigid and soft insoles and, eyes shut). Three variables were considered to assess stability: length of movement of the barycenter (mm), lateral velocity (mm/sg) and anterior velocity (mm/sg). All of them were analysed with the patient in static on the dynamometric platform. The results showed statistically significant differences between the two groups, (amputees and controls) with less stability in the amputee group (*p* < 0.05) when analysing the variables of length of movement of the barycenter, lateral velocity and anterior velocity. Amputee patients with open eyes exhibited greater stability than those with closed eyes. The hard insoles improved the stability data in amputees (length of movement of the barycenter and anterior velocity) with respect to the barefoot condition, and the soft insoles showed less stability than the patients with hard insoles, or than the barefoot patients. From the results obtained in this study, we can conclude that the PP-DWST 4 mm rigid insoles improve static stability in people with amputation. However, soft insoles impair stability and are therefore discouraged.

## Introduction

Postural stability is the ability to control the body position in space for the purpose of movement and balance^1^. It is necessary for maintaining a static position and for assisting body coordination in dynamic position changes^2^. Maintaining this posture entails a dynamic concept of posture where the control of neuromuscular activity is essential, as well as different variables that determine balance and stability^3^.

People with lower limb amputation show greater displacements of their centre of gravity in static situations than able-bodied individuals, depending to a greater extent on visual information^4^. In static balance, the ankle strategy is responsible for controlling displacements in the anterior–posterior axis by modulating the amount of torque developed by the plantar and dorsal ankle flexors. In people with lower limb (LL) amputation, the ability to use an ankle strategy is severely impaired, which would explain the instability in that direction^4^. As ambulation requires body movements primarily in the sagittal plane, it must be assumed that people with LL amputation will express greater difficulty in controlling and maintaining balance during gait. In fact, people with amputation have a higher risk of falling compared to age-matched able-bodied individuals (52% of individuals with transtibial amputation report at least one fall in a twelve-month period)^5,6^, This leads to additional health care costs, and a setback in the social and occupational adaptation of the patient with amputation.

Different studies indicate that lower limb amputees suffer from postural and gait alterations^7–9^. In this way Arya et al.^10^ studied three male patients with amputation aged between 43 and 47 years with dynamometric platforms, in order to assess the characteristics and evolution of gait in these patients. Jayakaran, Johnson, and Sullivan compared postural control of transtibial amputees of different amputation etiologies with healthy adults under altered sensory testing conditions^11–13^.

Other studies focus on different ankle and foot prostheses to improve balance^14^; on the limits of stability in people with transtibial amputation with respect to alterations of prosthesis alignment^15^; on the stability of people with transtibial traumatic amputation during standing on different terrain, including flat terrain and on slopes, etc.^16,17^.

However, no studies have been carried out on the influence of the use of insoles on the stability of amputee patients. It is known that a significant predictor of falls in the elderly population is attributed to postural instability. Shoe insoles have been identified as a mechanism to enhance postural control in older adults^18^. Insoles are considered cost-effective and easily adopted tools to improve balance. Numerous studies have verified their immediate effects on balance^19^. This leads us to hypothesize that the use of insoles can improve stability in amputee patients.

The aim of our study is to analyse and measure the influence of rigid and soft insoles on the instability of prosthetic transtibial amputees vs. able-bodied patients using a dynamometric platform in two situations: eyes open and shut; and to check whether the density of the insole material influences the maintenance of postural stability.

The static stability will be evaluated while barefoot rather than wearing shoes because footwear has been shown to affect sensory feedback, potentially acting as a sensory filter between the feet and the standing surface^18,20,21^.

Our purpose is to investigate the effects on the static stability of different insole materials in unilateral transtibial amputation and to implement practical clinical interventions in terms of stabilizing postural sway, avoiding possible falls and therefore implying a benefit for these patients.

## Methods

### Design

A prospective pre-post longitudinal quasi-experimental study was carried out. The protocol was verified and approved by the Research Ethics Committee of the Community of Aragon (CEICA) (Registration no: PI18/403), and all subjects signed an informed consent form prior to participating in the study.

The ethical principles for medical research in human subjects of the Declaration of Helsinki adopted at the 18th Assembly of the World Medical Association (WMA) (Helsinki, Finland, June 1964) were followed, amended at the 52nd General Assembly (Edinburgh, Scotland, October 2000), with note of clarification of paragraph 29 (WMA General Assembly, Tokyo 2004) revised at the 59th WMA General Assembly (Seoul, Korea, October 2008) and last revised version at the 64^th^ WMA General Assembly held in Fortaleza, (Brazil, October 2013)^22^.

### Participants

Two population groups were recruited through voluntary participation.

The first group comprised 25 unilateral transtibial amputee participants between 18 and 70 years old with prosthesis. Study participants included 4 participants with vascular transtibial amputation, 3 participants with diabetes, 11 participants with traumatic transtibial amputation, 3 participants with agenesis, 2 participants with oncological amputation and 2 participants with infection.

The inclusion criteria were that all subjects were free from musculoskeletal disorders and leg pain; they were a competent walker, which means they could carry out a standing ambulation by themselves without the help of third parties or the help of walking devices such as walking sticks. Each patient used his/her own prosthesis and had at least two years’ experience with the device to ensure that there was a correct adaptation (mean time since amputation was 15.1 ± 14.8 years).

A 7 cm transtibial amputation was assessed from the joint interline of the knee, being the optimal level of stump length 12 cm below the knee interarticular line.

The amputee participants did not have knee instability. The alignment of each prosthesis was checked by a technician before the test. All subjects used axial type prosthetic feet, and gave informed consent to the study. The prosthetic foot used for all amputees was the Vari-Flex® foot.

The second group comprised 25 control participants (control group) with no stability problems in standing position or mobility problems, and who gave their consent to the study. These were non-amputee participants between 18 and 70 years old, with similar height and weight to those of the amputee participants, and whose medical histories did not indicate any osteoarticular or neurological disorders. They went to the podiatric clinic for an occasional podiatric problem such as an incarnate toenail, hyperkeratosis or treatment of a plantar wart in foot.

The control participants were recruited from the Sarroca Podiatry Clinic, and the transtibial amputee individuals came from several entities: Asociación de Amputados Adampi, Ortopedia Axis, Ortopedia Alcalá, Ortopedia Zaraorto and different hospitals according to a random sampling method, after identifying the eligible population through a clinical examination by the principal investigator to determine inclusion.

### Baropodometric study

**Figure 1:**
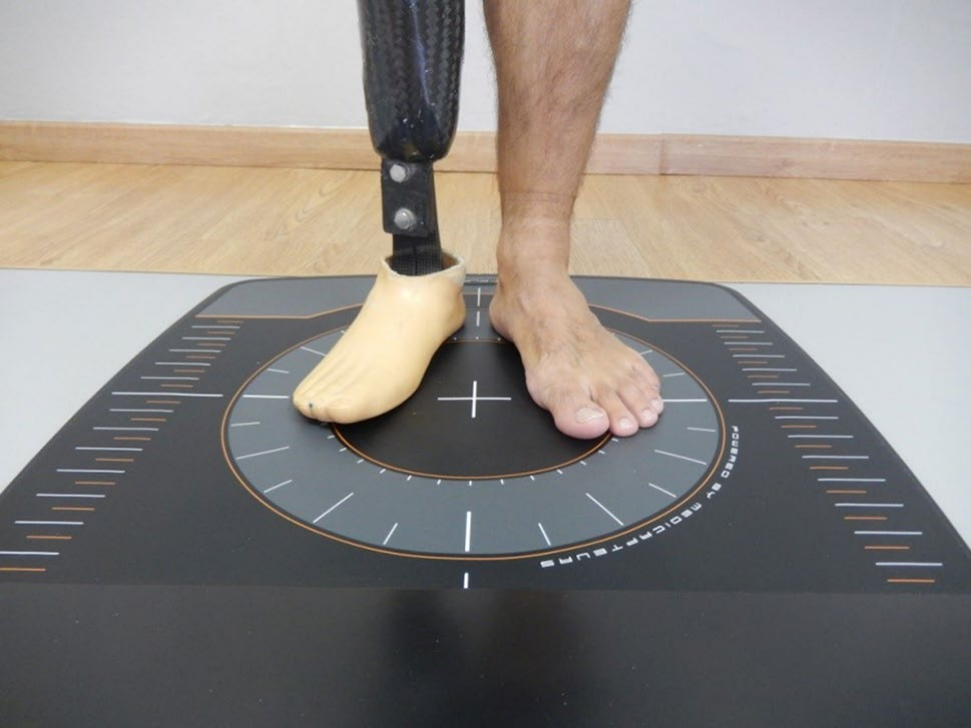
Baropodometry on a barefoot amputee patient in a comfortable bipedal position on the platform with both heels 2 cm apart with forefoot creating a 30° angle.

The baropodometric study (Fig. 1) was performed by the same clinician who performed the outcome measurements before and after the interventions with a Footwork force platform (V-PLACA, Standard EN 46,003; Medicapteurs, Balma, France)^23^. The Footwork force platform offers many parameters for assessing postural stability. For the present study, we have evaluated the most commonly used in scientific literature and those that have shown the greatest reliability in different population groups^24,25^. The characteristics of the Footwork dynamometric platform are specified in Table 1.

**Table 1:** Technical specifications of force platforms.

Size (length/width/height)	530 × 600 × 45 mm
Thickness	4 mm
Active surface	400 × 400 mm
Weight	6.8 kg
Sensors	Calibrated resistive
Sensor size	8 × 8 mm
Thickness sensor	0.15 mm
Number of sensors	2304 (48 × 48)
Permissible temperature	− 40 to 85 °C/ − 40 to 185 °F
Pressure min/max sensor	0.4 N/m^2^ = 0.0004 kPa / 100 N/m^2^ = 0.1 kPa
Type PC interface/platform	USB
Supply	USB cable
Data acquisition frequency	200 images / second
Vertical force recording	60 Hz
Operating system required	Windows XP, Vista, 7

Abbreviations: *mm* millimetres, *Kg* kilograms, *°C* degrees Celsius, *°F* degrees Fahrenheit, *N/m*^*2*^ Newton/square metre, *kPa* kilopascals, *Hz* Hertzios.

The system consists of a force platform placed on the floor. It was calibrated for the weight of each individual.

Data collection was performed with the subjects standing on the platform in a comfortable bipedal position according to standardised procedures: heels of the feet were 2 cm apart with the forefoot creating a 30° angle (Fig. 1). This ensured that the centre of gravity was positioned within a triangle formed by the foot support^26^.

A reference point was located in front of the patients, based on their height, and they were asked to keep their gaze fixed on the reference point and hold their position for 30 s. If the participant moved during the test, then the data were discarded and the test was repeated^26^.

Testing continued until data were obtained from 6 trials during which the participant was stationary. The participant did not know when he/she was actually being recorded. The average of 6 valid trials per foot was used for further analysis^26^.

The following data were collected for each patient in both groups: length of movement of the barycenter (mm), lateral velocity (mm/sg), and anterior velocity (mm/sg).

The center of gravity (COG) is considered to be the point where the entire weight of the body is concentrated. Stabilometry therefore measures the stability of individuals by reducing them to a point, being able to objectively define the average position of the COG, as well as the small movements that it may undergo around this position.

- Length of movement of the barycenter is the length traveled by the center of gravity; the length of the trajectory covered by the centre thrust, and is measured in millimeters (mm).
- Lateral velocity is the velocity of the displacement of the COG projection in the X axis in postural oscillations and is measured in mm/sg.
- Anterior velocity is the velocity of the displacement of the COG projection in the Y axis in the postural oscillations and is measured in mm/sg.

To examine the influence of altered somatosensory information on postural stability, the participants performed standing balance tests under three surface conditions:

1. Subject with bare feet.
2. Subject with hard textured surface insole. 4 mm rigid material: Polypropylene PP-DWST. Manufactured by SIMONA (D-55606 Kirn, Germany) and distributed in Spain by Al-Mar Técnicas Ortopédicas S.L. (Arganda del Rey, Madrid).
3. Subject with soft silicone comfort surface insole. Soft silicone material (Varisan^©^ hydrogel insoles, Farmavari S.A.U., Meres, Spain).

For each of the surface conditions, participants were tested in two vision conditions (eyes open, eyes shut) on three permanent surfaces (barefoot without insole, barefoot on hard insole and barefoot on soft insole). This was done barefoot because if we shoe the patient the Center of Gravity could be displaced depending on the drop of the sole or the height of the heel^10,24,27,28^.

According to previous research^24,25,27,28^, our study used measurements derived from centre of pressure (COP) displacement, and included the anterior and lateral velocity range.

The platform pressure sensor measurements were close to 0.001 kg/cm^2^. Before each use, automatic calibration was performed according to the manufacturer's instructions^29^.

For each situation, three measurements were performed, and the mean subsequently calculated in order to assess reliability. After a few days, the same variables were measured again to check repeatability^30^.

Data recording was performed with a personal computer linked to the pressure sensor platform. The commercially available S-plate software program version 7.0 (interactive rehabilitation software) for Windows (Medicapteurs, Balma, France) was used for all data collection and management.

### Statistical analysis

Continuous variables were expressed as mean with standard deviation (SD) whereas qualitative variables were expressed as frequencies and percentages. The Kolmogorov–Smirnov test was used to assess the normality of the quantitative variables under study.

To assess the reliability of the parameters within the same test day in each patient, the intraclass correlation coefficients (ICC) were used^31,32^. Using the classification proposed by Landis and Koch^31^, ICCs between 0.20 and 0.40 are considered to demonstrate reasonable reliability. Scores between 0.40 and 0.60 have moderate reliability, scores between 0.60 and 0.80 have considerable reliability, and in the highest category, scores ranging from 0.80 to 1.00 are considered near perfect. Other authors^33^ indicate that to obtain reliability, an ICC value of at least 0.75 must be obtained.

To assess the relationship between the study group (patients vs. controls) and the sociodemographic, clinical and stability variables, the Mann–Whitney test or Student's t-test was used for quantitative variables, according to normality criteria. In the case of qualitative variables, the Chi-square test or Fisher's test was used.

To compare the results of stability in the different situations evaluated (support, vision), methods of comparison of means for related samples were applied. Wilcoxon test for two situations and Friedman test for three or more when the variable does not follow a normal distribution, and Student's t-test or repeated measures ANOVA if the variable follows a normal distribution.

The significance level was set at *p* < 0.05. However, in order to address the issue of conducting multiple comparison, the Bonferroni correction was applied (*P*-value < 0.05/3 = *P*-value < 0.017).

Statistical analyses were performed using the statistical software IBM SPSS Statistics, version 26 (SPSS Ibérica, Madrid, Spain).

### Informed consent statement

Informed consent was obtained from all subjects involved in the study.

## Results

### Sociodemographic characteristics attending to the division by treatment groups

We had 50 participants in the study, 25 controls and 25 participants with amputation. In each of the groups, 80.0% of the subjects were men (20/25) and 20.0% women (5/25). The average age of the patients with amputation was 44.0 ± 12.9 years and that of controls was 38.4 ± 12.4 years. This difference between the groups was not statistically significant (*p* = 0.124). The average body mass index (BMI) of individuals with amputation was 26.4 ± 4.8 kg/m2 and that of controls 25.0 ± 3.1 kg/m2. This difference between the groups was not statistically significant (*p* = 0.220) (Table 2).

**Table 2:** Sociodemographic characteristics of participants by study group.

Variable	Total *n* = 50	Control Group *n* = 25	Amputee Group *n* = 25	*p* value*
Male	20 (80.0%)	20 (80.0%)	20 (80.0%)	1.000
Average age (years) ± SD	41.2 ± 12.9	38.4 ± 12.4	44.0 ± 12.9	0.124
Average BMI (kg/m^2^) ± SD	25.7 ± 4.0	25.0 ± 3.1	26.4 ± 4.8	0.220

*BMI* Body Mass Index, *SD* standard deviation.

*Mann–Whitney Test.

### Length of movement of the barycenter

Table 3 shows the descriptive parameters of the variable “*Length of movement of the barycenter”* for the six situations evaluated, showing statistically significant differences (*p* < 0.05) between the two groups, with patients with amputation obtaining higher average levels than controls.

**Table 3:** Comparison of Length of movement of the barycenter between experimental groups.

	Control group	Amputee group	Difference	Sig.^1^ (*p*)
**Barefoot**				
Eyes open	64.8 (20.4)	120.1 (46.3)	55.3 (10.1)	** < 0.001**
Eyes shut	84.9 (31.1)	274.7 (181.1)	189.8 (36.8)	** < 0.001**
**Soft insole**				
Eyes open	80.6 (28.2)	126.8 (66.0)	46.2 (14.4)	**0.001**
Eyes shut	116.7 (61.3)	292.2 (171.7)	175.5 (36.5)	** < 0.001**
**Hard insole**				
Eyes open	58.2 (25.3)	116.2 (74.6)	58.0 (15.8)	** < 0.001**
Eyes shut	80.9 (38.1)	228.9 (136.3)	148.0 (28.3)	** < 0.001**
Sig^2^ (*p*)	** < 0.001**	** < 0.001**		

Significant values are in [bold].

Mean (standard deviation).

^1^Mann–Whitney Test.

^2^Friedman Test.

Length of movement of the barycenter (mm).

Annex 1 shows the *p* values of the two-to-two comparisons. The differences were statistically significant when comparing open eyes vs closed eyes for the three supports (barefoot, soft footbed, hard footbed) in both groups.

In addition, in the control group, significant differences (*p* < 0.05) were observed between soft insole and barefoot or soft insole and hard insole (at the same vision condition), also between hard and barefoot for open eyes. In amputee patients, there were differences between the supports in relation to the average length of movement of the barycenter only for closed eyes.

### Length of movement of the barycenter according to support

Table 4 shows the descriptive parameters of the variable “*Length of movement of the barycenter”* for the three supports: barefoot, soft insole and hard insole. The data are grouped into eyes open and eyes shut.

**Table 4:** Comparison of length of movement of the barycenter between the different insoles in each experimental group.

	Control group	Amputee group	Difference	Sig.^2^ (*p*)
**Barefoot**				
	74.8 (24.8)	197.4 (103.6)	122.5 (21.3)	** < 0.001**
**Soft insole**				
	98.6 (43.4)	209.5 (111.7)	110.9 (24.0)	** < 0.001**
**Hard insole**				
	69.5 (30.6)	172.5 (95.2)	103.0 (20.0)	** < 0.001**
Sig^1^. (*p*)	** < 0.001**	**0.006**		

Significant values are in [bold].

Mean (standard deviation) [IC. For the mean (at 95%)]. ^1^Friedman Test. ^2^Mann-Whitney Test.

Length of movement of the barycenter (mm).

Statistically significant differences were observed between the three supports in the two groups (*p* < 0.05), with greater stability (shorter length of movement of the barycenter) with the hard insole.

Hard insole, barefoot and soft insole would be the order of the three supports from most to least stable, both for people with amputation and controls.

In the group of amputees, the hard insole obtained significantly lower length of movement of the barycenter values than barefoot (*p* = 0.010), but no differences were observed between barefoot and soft insole (*p* = 0.925). In the control group this was different, the hard insole did not show values significantly lower than barefoot (*p* = 0.061), but there were differences between barefoot and soft insole (*p* < 0.001). Annex 2 shows the p values of the two-to-two comparisons.

### Lateral velocity

Table 5 shows the descriptive parameters of the variable "*Lateral velocity*" for the six situations evaluated, showing statistically significant differences (*p* < 0.05) between the two groups for all of them, with people with amputation obtaining higher levels on average than controls.

**Table 5:** Comparison of lateral velocity between experimental groups.

	Control group	Amputee group	Difference	Sig.^1^ (*p*)
**Barefoot**				
Eyes open	1.4 (0.5)	2.7 (0.9)	1.3 (0.2)	** < 0.001**
Eyes shut	1.7 (0.5)	5.6 (3.3)	3.9 (0.7)	** < 0.001**
**Soft insole**				
Eyes open	1.7 (0.5)	2.8 (1.4)	1.1 (0.3)	** < 0.001**
Eyes shut	2.4 (1.3)	6.1 (3.5)	3.7 (0.7)	** < 0.001**
**Hard insole**				
Eyes open	1.2 (0.6)	2.6 (1.7)	1.4 (0.4)	** < 0.001**
Eyes shut	1.6 (0.7)	4.9 (2.8)	3.3 (0.6)	** < 0.001**
Sig^2^ (*p*)	** < 0.001**	** < 0.001**		

Significant values are in [bold].

Mean (standard deviation). ^1^Mann–Whitney Test. ^2^Friedman Test.

Lateral velocity (mm/sg).

Annex 3 shows the p values for each of the two-to-two comparisons. The differences are statistically significant when comparing open eyes vs closed eyes for the three supports (barefoot, soft insole, hard insole) in amputee patients and in controls.

Furthermore, in the control group, it is worth noting the significant differences (*p* < 0.05) between hard insole and soft insole, regardless of the vision condition.

### Lateral velocity according to support

Table 6 shows the descriptive parameters of the variable "*Lateral velocity*" for the three supports: barefoot, soft insole and hard insole, grouping the data into eyes open and eyes shut.

**Table 6:** Comparison of lateral velocity between the different insoles in each experimental group.

	Control group	Amputee group	Difference	Sig.^2^ (p)
**Barefoot**				
	1.6 (0.5)	4.2 (1.9)	2.6 (0.4)	** < 0.001**
**Soft insole**				
	2.1 (0.9)	4.4 (2.3)	2.3 (0.5)	**< 0.001**
**Hard insole**				
	1.4 (0.6)	3.8 (2.1)	2.4 (0.4)	**< 0.001**
Sig^1^. (p)	** < 0.001**	0.125		

Significant values are in [bold].

Mean (standard deviation). ^1^Friedman Test. ^2^Mann–Whitney Test.

Lateral velocity (mm/sec).

Statistically significant differences between the three supports are observed in the control group, with greater stability (lower lateral velocity) with the hard insole. However, in the amputee group there are no significant differences in the lateral velocity of the patients in the different types of support (*p* = 0.125).

Hard insole, barefoot and soft insole would be the order of the three supports from the highest to the lowest stability for both groups (Table 6).

Statistically significant differences were observed between the three supports in the control group, presenting greater stability (lower lateral velocity) with a hard insole. Hard insole, barefoot and soft insole would be the order of the three supports from highest to lowest stability for controls; the three situations show statistically significant differences in comparisons two to two (Annex 4). In the group of amputees, the hard template obtained significantly lower lateral velocity values than the soft template (*p* = 0.005). Although the mean value of lateral velocity shows the same trend in amputees, there are no significant differences between the three types of support in this group.

### Anterior velocity

Table 7 shows the descriptive parameters of the variable "*Anterior velocity"* for the six situations evaluated, showing statistically significant differences (*p* < 0.05) between the two groups for all of them, with people with amputation obtaining higher average levels than controls.

**Table 7:** Comparison of anterior velocity between experimental groups.

	Control group	Amputee group	Difference	Sig.^1^ (*p*)
**Barefoot**				
Eyes open	1.4 (0.5)	2.5 (1.1)	1.1 (0.2)	** < 0.001**
Eyes shut	1.9 (0.8)	6.1 (4.5)	4.2 (0.9)	** < 0.001**
**Soft insole**				
Eyes open	1.7 (0.7)	2.6 (1.4)	0.9 (0.3)	**0.003**
Eyes shut	2.5 (1.4)	6.3 (3.9)	3.8 (0.8)	** < 0.001**
**Hard insole**				
Eyes open	1.3 (0.5)	2.4 (1.5)	1.1 (0.3)	** < 0.001**
Eyes shut	1.9 (1.0)	4.9 (3.1)	3.0 (0.7)	** < 0.001**
Sig^2^ (*p*)	** < 0.001**	** < 0.001**		

Significant values are in [bold].

Mean (standard deviation). ^1^Mann–Whitney Test. ^2^Friedman Test.

Anterior velocity (mm/sec).

Annex 5 shows the p values for each of the two-to-two comparisons. The differences are statistically significant when comparing open eyes vs closed eyes for the three supports (barefoot, soft insole, hard insole) in both groups.

Furthermore, in the control group, it is worth noting the significant differences (*p* < 0.05) between hard insole and soft insole, regardless of the vision condition. In the group of patients, hard insole shows significant differences with barefoot and soft insole with eyes open.

### Anterior velocity according to support

Table 8 shows the descriptive parameters of the variable "*Anterior velocity*" for the three supports: barefoot, soft insole and hard insole, grouping the data into eyes open and eyes hut.

**Table 8:** Comparison of anterior velocity between the different insoles in each experimental group.

	Control Group	Amputee Group	Difference	Sig.^2^ (*p*)
**Barefoot**				
	1.6 (0.7)	4.3 (2.6)	2.7 (0.5)	** < 0.001**
**Soft insole**				
	2.1 (1.0)	4.5 (2.4)	2.4 (0.5)	** < 0.001**
**Hard insole**				
	1.6 (0.7)	3.6 (2.1)	2.0 (0.4)	** < 0.001**
Sig^1^. (*p*)	** < 0.001**	**0.013**		

Significant values are in [bold].

Mean (standard deviation) [IC. For the mean (at 95%)]. ^1^Friedman Test.^ 2^Mann-Whitney Test.

Anterior velocity (mm/sec).

Statistically significant differences were observed between the three supports in both study groups (*p* < 0.05), with greater stability (lower anterior velocity) with the hard insole. Hard insole, barefoot and soft insole would be the order of the three supports from greater to lesser stability.

Annex 6 shows the p values of the two-to-two comparisons. In the group of amputees, the hard insole obtained significantly lower anterior velocity values than barefoot (*p* = 0.012) and soft insole (*p* = 0.001), but no differences were observed between barefoot and soft insole (*p* = 0.914). In the control group, however, the hard insole did not show significantly lower values than barefoot (*p* = 0.158), but there were differences between barefoot and soft insole (*p* < 0.001) and between hard insole and soft insole (*p* < 0.001).

### Reliability analysis

The measurements showed good/acceptable reliability with CI levels of above 0.79 for all stability variables considered, thus justifying the use of the mean value for the data analysis. The specific CI values are provided in Annex A.

## Discussion

Taking into account the “length of movement of the barycenter” parameter, which projects the amplitude of the centre of gravity in millimetres, patients with amputation show less stability (higher mean levels) than controls. There are differences between the proposed situations (support and vision), obtaining greater stability (shorter length of movement of the barycenter) with eyes open compared to eyes shut, and with hard insole compared to barefoot or soft insole (less stable situation), both in people with amputation and controls.

In our study, people with lower limb amputation present greater displacements of their centre of gravity in a static situation than able-bodied individuals, depending to a greater extent on visual information. These data are similar to those of other studies carried out on 6 people with amputation and 6 controls in 2002^4^. Considering the “lateral velocity” parameter, people with amputation show less stability (higher mean levels) than controls. In people with amputation, there are differences between the two proposed vision conditions, obtaining greater stability (lower lateral velocity) with eyes open compared to eyes shut. However, the differences according to support are not significant in this group in terms of lateral velocity.

Taking into account the “anterior velocity” parameter, people with amputation show less stability (higher mean levels) than controls. There are differences between the proposed situations (support and vision) in both people with amputation and controls, obtaining greater stability (lower anterior velocity) with eyes open compared to eyes shut, and with hard insole compared to barefoot or soft insole. In this case, in static balance, the ankle strategy is in charge of controlling the displacements in the anterior–posterior axis, modulating the amount of “torque” developed by the plantar and dorsal ankle flexors. In people with LL, the ability to use an ankle strategy is severely impaired, which would explain the greater instability in this direction compared to the lateral direction^4^.

There are studies that indicate that individuals with unilateral transtibial amputation would have serious difficulties in bearing the load on the lower limb as a consequence of the joint instability that seems to exist in the sagittal and frontal planes, essentially at the level of the knee joint^34^.

Despite improved prosthetic components, the most recent studies still show asymmetries in the load between the two lower limbs of the individuals with amputation^35–37^. As a result of this asymmetry, the healthy lower limb is continuously subjected to high mechanical stresses, which can lead to pain or degeneration of the articular cartilage^38,39^.

The visual system shows great importance in the control of balance in all the subjects studied, both those with amputations and those belonging to the control group, as in all the variables and situations studied, better stabilometric results are obtained with eyes open as opposed to eyes shut.

There are studies that state that it is sufficient for the subject to close their eyes for their oscillations to increase by 250%^40^.

In 2000, Fransson et al.^41^ concluded that all individuals change tactics when they close their eyes, with the control of postural oscillations being much less precise. Similarly, in our study, an increase in postural oscillations can be observed when vision with eyes shut is eliminated.

Consistent with our results, Buckley et al.^4^ state that people with lower limb amputation show greater displacements of their centre of gravity in a static situation than controls, depending to a greater extent on visual information. People with amputation had a greater problem controlling dynamic balance in the anterior–posterior direction than in the mediolateral direction.

Therefore, like Arifin et al.^42^, the results obtained recognise proprioception as an important quality for maintaining stability and postural strategy depending on the different support surfaces. They also point out that distorted somatosensory inputs are among the problems leading to impaired balance control in people with amputation. They further mention that reduced proprioception is associated with asymmetry in load bearing and decreased confidence in people with amputation. In the present study, this is reflected in the difference between eyes open and shut on the pressure platform to assess stability. Arifin et al.^42^ state that flexible or unstable surfaces reduce the ability to accurately detect body orientation, which is fully consistent with the findings presented in our study.

However, other studies show controversial results in relation to visual inflow and postural stability in people with amputations.

Nevertheless, Vittas et al. tested 20 people with transtibial amputation and concluded that their vertical axis stability was as good as the group of able-bodied individuals^43^.

These contradictory results between researchers could be due to the different methods used to assess stability.

To our knowledge, no previous investigations have described the effects of insole density on the stability of amputee patients, but the use of different devices to increase stability has been addressed in other vulnerable populations such as older adults. Finlay et al. reported that foot function and stability is enhanced when subjects wear a prescribed shoe (orthopaedic) compared with values obtained when subjects wear their own footwear in this population^18,44^. In fact, shoe insoles have also been identified as a mechanism to enhance postural control because they improved somatosensory function and may be useful in balance control^45^. Changes in muscle activity as a consequence of various insole densities suggest an enhancement of plantar surface sensory input^18,19^, which is important since the maintenance of postural stability is dependent on a range of somatosensory inputs. Shoe insoles can increase plantar foot surface contact and potentially increase somatosensory input. Regarding the influence of insole material in amputee patients, Sarroca et al. described an increase in thigh muscle activity recorded by electromyography studies in this vulnerable population^46^.

Losa et al. reported significant improvements in postural sway when older adults stood on both soft and hard insoles compared to standing barefoot^18^. However, our results exhibit that the soft insole shows the most unstable situation in the amputee group and in the group of able-bodied patients, followed by barefoot patients. The most stable situation in both groups in our study (healthy and amputees) is on the rigid PP-DWST insole which is consistent with the results provided by Losa et al. in which they also found more pronounced improvements when a hard insole was used in older adults^18^.

It is worth highlighting the importance of the sample size in our study (25 patients with amputation, 25 controls) compared to others, which demonstrates the reliability of the results and the incorporation of new lines of research with plantar support rigidity in stabilometry.

## Conclusion

After evaluating postural stability by means of stabilometry under normal conditions (on the floor of the dynamometric platform with eyes open), and under altered conditions (with the interposition of different materials such as plantar support and shut eyes) in two groups (25 patients with amputation and 25 able-bodied individuals), we can conclude that subjects with unilateral transtibial amputation present less stability in terms of length of movement of the barycenter, lateral velocity and anterior velocity than the able-bodied population. Greater stability is obtained in both groups with eyes open compared to eyes shut, and the length of movement of the barycenter and anterior velocity in both groups is significant (more stability) with hard insole compared to barefoot or soft insole. However, in the group of people with amputation there are no significant differences in the lateral velocity of the patients in the different types of support. Providing increased sensory inputs with hard insoles may be an effective way to reduce the rate of falls, which are related to decreased quality of life in amputee individuals. This study can aid in better understanding the insole design features associated with improved postural stability in amputee individuals.

## Supplementary Information

Supplementary Information 1.

Supplementary Information 2.
