# Supplementary material for: Influence of insole material density in the stability of patients with prosthetic unilateral transtibial amputation
Source: Sci Rep. 2022 May 12;12:7854. doi: 10.1038/s41598-022-11564-3 (PMC9098549; doi:10.1038/s41598-022-11564-3)
Supplement: Supplementary file 1 — Supplementary Information 1. [file 41598_2022_11564_MOESM1_ESM.docx]

Annex A: Table of ICC reliability (95%)of the three repeated measurements of the stability variables.

| **Variable** | **ICC** | **Limit** | **Limit** |
| --- | --- | --- | --- |
| LENGTH_DS_OPEN | 0.845 | 0.767 | 0.903 |
| LENGTH_DS_SHUT | 0.895 | 0.838 | 0.940 |
| LENGTH_B_OPEN | 0.833 | 0.749 | 0.895 |
| LENGTH_B_SHUT | 0.912 | 0.864 | 0.946 |
| LENGTH_D_OPEN | 0.826 | 0.739 | 0.890 |
| LENGTH_D_SHUT | 0.939 | 0.905 | 0.963 |
| LATERALV_DS_OPEN | 0.815 | 0.724 | 0.883 |
| LATERALV_DS_SHUT | 0.870 | 0.803 | 0.919 |
| LATERALV_B_OPEN | 0.823 | 0.735 | 0.888 |
| LATERALV_B_SHUT | 0.893 | 0.837 | 0.934 |
| LATERALV_D_OPEN | 0.843 | 0.764 | 0.902 |
| LATERALV_D_SHUT | 0.922 | 0.897 | 0.952 |
| ANTERIORV_DS_OPEN | 0.848 | 0.771 | 0.905 |
| ANTERIORV_DS_SHUT | 0.896 | 0.841 | 0.936 |
| ANTERIORV_B_OPEN | 0.804 | 0.709 | 0.875 |
| ANTERIORV_B_SHUT | 0.914 | 0.868 | 0.947 |
| ANTERIORV_D_OPEN | 0.791 | 0.690 | 0.867 |
| ANTERIORV_D_SHUT | 0.936 | 0.899 | 0.961 |
