# Supplementary material for: Influence of insole material density in the stability of patients with prosthetic unilateral transtibial amputation
Source: Sci Rep. 2022 May 12;12:7854. doi: 10.1038/s41598-022-11564-3 (PMC9098549; doi:10.1038/s41598-022-11564-3)
Supplement: Supplementary file 2 — Supplementary Information 2. [file 41598_2022_11564_MOESM2_ESM.docx]

ANNEXES

**Annex 1** Length of movement of the barycenter according to situation and study group (upper diagonal amputees-lower diagonal controls). Two-to-two comparisons.

| **Length of movement of the barycenter** | **Barefoot**  **Open eyes** | **Barefoot**  **Closed eyes** | **Soft Insole**  **Open eyes** | **Soft Insole**  **Closed eyes** | **Hard Insole Open eyes** | **Hard Insole Closed eyes** |
| --- | --- | --- | --- | --- | --- | --- |
| **Barefoot**  **Open eyes** |  | **<0,001** | 0,904 | **<0,001** | 0,088 | **<0,001** |
| **Barefoot**  **Closed eyes** | **<0,001** |  | **<0,001** | 0,600 | **<0,001** | **0,048** |
| **Soft Insole**  **Open eyes** | **<0,001** | 0,242 |  | **<0,001** | 0,060 | **<0,001** |
| **Soft Insole**  **Closed eyes** | **<0,001** | **<0,001** | **<0,001** |  | **<0,001** | **0,001** |
| **Hard Insole**  **Open eyes** | **0,040** | **<0,001** | **<0,001** | **<0,001** |  | **<0,001** |
| **Hard Insole**  **Closed eyes** | **0,011** | 0,247 | 0,619 | **<0,001** | **<0,001** |  |

**Annex 2** Length of movement of the barycenter according to support and study group (upper diagonal amputees-lower diagonal controls). Two-to-two comparisons.

| **Length of movement of the barycenter** | **Barefoot** | **Soft Insole** | **Hard Insole** |
| --- | --- | --- | --- |
| **Barefoot** |  | 0,925 | **0,010** |
| **Soft Insole** | **<0,001** |  | **0,001** |
| **Hard Insole** | 0,061 | **<0,001** |  |

**Annex 3** Lateral velocity according to situation and study group (upper diagonal amputees-lower diagonal controls). Two-to-two comparisons.

| **Lateral velocity** | **Barefoot**  **Open eyes** | **Barefoot**  **Closed eyes** | **Soft Insole**  **Open eyes** | **Soft Insole**  **Closed eyes** | **Hard Insole Open eyes** | **Hard Insole Closed eyes** |
| --- | --- | --- | --- | --- | --- | --- |
| **Barefoot**  **Open eyes** |  | **<0,001** | 0,954 | **<0,001** | **0,041** | **<0,001** |
| **Barefoot**  **Closed eyes** | **<0,001** |  | **<0,001** | 0,581 | **<0,001** | 0,093 |
| **Soft Insole**  **Open eyes** | **0,001** | 0,819 |  | **<0,001** | 0,100 | **<0,001** |
| **Soft Insole**  **Closed eyes** | **<0,001** | **<0,001** | **<0,001** |  | **<0,001** | **0,003** |
| **Hard Insole**  **Open eyes** | **0,026** | **<0,001** | **<0,001** | **<0,001** |  | **<0,001** |
| **Hard Insole**  **Closed eyes** | 0,211 | 0,106 | 0,072 | **<0,001** | **<0,001** |  |

**Annex 4** Lateral velocity according to support and study group (upper diagonal amputees-lower diagonal controls). Two-to-two comparisons.

| **Lateral velocity** | **Barefoot** | **Soft Insole** | **Hard Insole** |
| --- | --- | --- | --- |
| **Barefoot** |  | 0,830 | 0,069 |
| **Soft Insole** | **<0,001** |  | **0,005** |
| **Hard Insole** | **0,036** | **<0,001** |  |

**Annex 5** Anterior velocity according to situation and study group (upper diagonal amputees-lower diagonal controls). Two-to-two comparisons.

| **Anterior velocity** | **Barefoot**  **Open eyes** | **Barefoot**  **Closed eyes** | **Soft Insole**  **Open eyes** | **Soft Insole**  **Closed eyes** | **Hard Insole Open eyes** | **Hard Insole Closed eyes** |
| --- | --- | --- | --- | --- | --- | --- |
| **Barefoot**  **Open eyes** |  | **<0,001** | 0,638 | **<0,001** | 0,183 | **<0,001** |
| **Barefoot**  **Closed eyes** | **<0,001** |  | **<0,001** | 0,757 | **<0,001** | **0,027** |
| **Soft Insole**  **Open eyes** | **<0,001** | **0,014** |  | **<0,001** | 0,170 | <0,001 |
| **Soft Insole**  **Closed eyes** | **<0,001** | **<0,001** | **<0,001** |  | **<0,001** | **0,001** |
| **Hard Insole**  **Open eyes** | 0,075 | **<0,001** | **<0,001** | **<0,001** |  | **<0,001** |
| **Hard Insole**  **Closed eyes** | **0,001** | 0,338 | 0,407 | **<0,001** | **<0,001** |  |

**Annex 6** Anterior velocity according to support and study group (upper diagonal amputees-lower diagonal controls). Two-to-two comparisons.

| **Lateral velocity** | **Barefoot** | **Soft Insole** | **Hard Insole** |
| --- | --- | --- | --- |
| **Barefoot** |  | 0,914 | **0,012** |
| **Soft Insole** | **<0,001** |  | **0,001** |
| **Hard Insole** | 0,158 | **<0,001** |  |
